# Inhibiting the Activity of CA1 Hippocampal Neurons Prevents the Recall of Contextual Fear Memory in Inducible ArchT Transgenic Mice

**DOI:** 10.1371/journal.pone.0130163

**Published:** 2015-06-15

**Authors:** Masanori Sakaguchi, Karam Kim, Lily Mae Yee Yu, Yoshiko Hashikawa, Yukiko Sekine, Yuki Okumura, Masako Kawano, Masanobu Hayashi, Deependra Kumar, Edward S. Boyden, Thomas J. McHugh, Yasunori Hayashi

**Affiliations:** 1 Brain Science Institute, RIKEN, Wako, Saitama, Japan; 2 International Institute for Integrative Sleep Medicine (WPI-IIIS), University of Tsukuba, Tsukuba, Japan; 3 The MIT Media Laboratory, Synthetic Neurobiology Group, Massachusetts Institute of Technology (MIT), Cambridge, Massachusetts, United States of America; 4 Saitama University Brain Science Institute, Saitama University, Saitama, Japan; Nathan Kline Institute and New York University Langone Medical Center, UNITED STATES

## Abstract

The optogenetic manipulation of light-activated ion-channels/pumps (i.e., opsins) can reversibly activate or suppress neuronal activity with precise temporal control. Therefore, optogenetic techniques hold great potential to establish causal relationships between specific neuronal circuits and their function in freely moving animals. Due to the critical role of the hippocampal CA1 region in memory function, we explored the possibility of targeting an inhibitory opsin, ArchT, to CA1 pyramidal neurons in mice. We established a transgenic mouse line in which tetracycline trans-activator induces ArchT expression. By crossing this line with a CaMKIIα-tTA transgenic line, the delivery of light via an implanted optrode inhibits the activity of excitatory CA1 neurons. We found that light delivery to the hippocampus inhibited the recall of a contextual fear memory. Our results demonstrate that this optogenetic mouse line can be used to investigate the neuronal circuits underlying behavior.

## Introduction

Optogenetics have become a popular technique for demonstrating a causal relationship between the function of a specific neuronal circuit and behavior [1–8]. The use of light-activated ion channels (i.e., opsins) in combination with other genetic tools allows for temporally precise, reversible, and selective manipulation of target neuron activity. This technique has begun to challenge conventional views of brain regions responsible for memory storage and retrieval and the temporal nature of their involvement in memory [9]. The primary advantage of optogenetic techniques is their ability to overcome many limitations inherent in older techniques. For instance, microinjections of reagents do not permit temporally precise control of neuronal activity such as spiking or allow the restriction of reagent effects to defined time windows or particular cell types. Similarly, electrical stimulation affects all neurons and axons within the vicinity of the electrode and cannot be used to inhibit neurons.

The use of inhibitory opsins, in particular, has begun to highlight the importance of temporally precise neuronal activity for memory function. For example, Goshen et al. showed the real-time involvement of hippocampal CA1 excitatory neurons during the acquisition and recall of recent contextual fear memory [1]. Gu et al. clarified the critical time point when adult-born hippocampal neurons most efficiently encode contextual and spatial memory [4]. Denny et al. and Tanaka et al. showed evidence that the deactivation of hippocampal neurons activated during learning is necessary for the retrieval of the memory [10,11]. Furthermore, other studies using inhibitory opsins show the importance of brain regions other than hippocampus in various stages of memory [2,3,5].

Two types of inhibitory opsins—archaerhodpsin (Arch; light-driven outward proton pump) and halorhodopsin (NpHR; inward chloride transporter)—have been widely used in neuroscience research [12–14]. Both opsins produce nA-scale photocurrents upon light stimulation, thereby generating reversible membrane hyperpolarization with step-like kinetic stability. However, after the cessation of an extended period of photo-activation, as is often required for studies of learning and memory, NpHR causes a rebound increase in the probability of synapse-evoked spiking through changes in the reversal potential of GABA_A_ receptors [15]. Therefore, Arch-based optogenetics may be a good alternative to NpHR-based techniques in certain experimental settings [16].

A challenge in utilizing optogenetic approaches in neuroscience research is the need to express high levels of opsin in each neuron due to the relatively small current mediated by each channel or pump [17,18]. Previous studies often used viral vectors to deliver opsins to target brain regions, resulting in incomplete coverage of the target region and variable expression levels between neurons and between animals. The use of transgenic, tetracycline-controlled transcriptional activation systems, however, allows opsins to be reversibly expressed in genetically defined cell populations by turning their expression on and off via the application of tetracycline or its derivatives (e.g., doxycycline) through the animal’s diet. Although some studies have applied similar approaches to express Arch [19] in the brain [10,11,17,20], to our knowledge, ArchT, a more sensitive version of Arch, has not previously been used to generate an inducible transgenic animal to study memory.

Here, we generated a TetO-ArchT mouse line in which ArchT is expressed in a defined cell population through its cross with an appropriate driver line. Using these mice, we confirmed that light delivery reliably inhibits CA1 neuronal activity *in vivo*. Furthermore, we showed that the recall of a fear memory in TetO-ArchT mice is reversibly inhibited by light delivery to the hippocampus, providing evidence of the utility of this new mouse line in memory research.

## Materials and Methods

### Animals

All experiments were ethically conducted in accordance with the Science Council of Japan’s Guidelines for Proper Conduct of Animal Experiments. Experimental protocols were approved by the Animal Care and Use Committees at the University of Tsukuba and RIKEN Brain Science Institute.

### Generation of TetO-ArchT transgenic mice

To generate TetO-ArchT-green fluorescent protein (GFP) mice, the ArchT-GFP cDNA fragment was subcloned to a pTRE-Tight (Clontech) vector with an additional SV40 polyA sequence amplified from a pMSG vector (Pharmacia) as a template in front of the existing SV40 polyA signal. Linearized DNA was injected into fertilized eggs from C57BL/6J mice (Support Unit for Animal Resources Development, RIKEN). Founders were bred with C57BL/6J mice to produce stable TetO-ArchT transgenic lines. A total of three TetO-ArchT transgene-positive founders were obtained, which we crossed with α-CaMKII-tTA (line B) mice [21] (The Jackson Laboratory).

### Optrode construction and surgical and recording procedures

Each optrode consisted of two core components: 1) an optic fiber for light delivery (200-μm diameter) and 2) nichrome tetrodes for extracellular recording of endogenous and light-inhibited single-unit responses in the CA1. Typically, two tetrodes (located approximately 180 μm apart) were coupled to the shaft of the optic fiber. The tip of each electrode protruded 200–400 μm from the end of the optic fiber and enabled the evaluation of spikes and local field potentials from neurons in the presence or absence of light. Each optic fiber and tetrode bundle could be advanced along the Z-axis via a mechanical screw.

To allow coupling of the optic fiber with the corresponding patch cord connected to the light source, a ceramic ferrule (1.25-mm diameter, Precision Fiber Products, Inc) was affixed to each optic fiber using epoxy glue. The ferrule of the optic fiber was coupled to an identical ferrule on the patch cord via a ceramic ferrule sleeve (Precision Fiber Products). Index matching gel (Thorlabs) was applied to the cleaned glass interface of the optic fiber and patch cord to aid optical coupling.

The optrode was surgically implanted in avertin-anaesthetized mice through a small craniotomy above the target recording site (relative to bregma: 1.70 mm posterior, 1.25 mm lateral). The optic fiber was placed in the superficial cortex during surgery. Mice were allowed to recover for ~2–4 days prior to advancement of the optrode toward the CA1 pyramidal cell layer. At the start of each recording session, the optic fiber was coupled to a patch cord (200 μm, Doric Lenses), and tetrodes were electrically connected to the preamplifier (Neuralynx) via an electrode interface board.

For optical inhibition experiments, the optic fiber was coupled to a 532-nm laser diode (Shanghai Lasers, ~20-mW fiber output). The optrode was slowly advanced through cortical layers and stratum oriens of the hippocampus until single-unit activity and high frequency ripple waveforms were visualized. Single units were manually clustered using Spikesort3D as previously described [22,23]. Quantification of baseline and light-induced responses was performed in Neuroexplorer and Matlab using in-house generated scripts.

We identified suppressed, non-responding, and stimulated neurons based on laser stimulation-induced changes in neuronal firing rate relative to baseline activity. Neurons showing a decrease or increase in activity greater than 2 standard errors (SE) were classified as suppressed or stimulated neurons, respectively, whereas those showing changes in activity less than 2 SE were classified as non-responding neurons.

### Optic cannula implantation for behavior study

For fear conditioning experiments, optic cannulae were constructed by gluing an optical fiber (200-μm core, multimode, 0.48 NA, Thorlabs) into an internal cannula (inner diameter 230-μm, outer diameter 1.25-mm, Plastics One). The fiber extended 5 mm beyond the cannula end. Two optic cannulae were placed bilaterally above the pyramidal cell layer of the CA1 region (relative to bregma: 2.30 mm posterior, ±1.6 mm lateral, 1.4 mm ventral). The other ends of the cannulae were connected to optic cables and attached to a 1:1 splitter/commutator (Doric Lenses), allowing bilateral illumination. The single end of the splitter was attached to a laser source (200 mW, 532 nm). Light output was adjusted to 10 mW at the fiber tip as measured by an optical power meter. Based on measurements from mammalian brains [24], light output of 10 mW at the fiber tip will produce ~3 mW/mm^2^ of light, which is sufficient to induce ArchT activation (~400 pA photocurrent) up to 1 mm directly away from the fiber tip [19,25].

### Contextual fear conditioning

Contextual fear conditioning was performed as previously described [26]. Briefly, the training and test context consisted of a stainless steel conditioning chamber (31 × 24 × 21 cm; MED Associates) containing a stainless steel shock grid floor. Shock grid bars (diameter, 3.2 mm) were spaced 7.9 mm apart. The grid floor was positioned over a stainless-steel drop pan, which was lightly cleaned with 70% ethyl alcohol to provide a background odor. The front, top, and back of the chamber were made of clear acrylic, and the sides were made of modular aluminum. Freezing was assessed using an automated scoring system (Actimetrics), which digitized the video signal at 4 Hz and compared movement frame-by-frame to determine the presence of freezing. During training, mice were placed in the context and after 2 min were presented with a 2-s footshock (0.5 mA). Mice remained in the context for an additional 30 s before being returned to their home cage. During the first test, the laser stimulation started when mice were placed into the chamber and ended when mice were removed from the chamber.

### Imaging and *in situ* hybridization

Sections were cut with a cryostat (Leica) at 50 μm thickness. The PCR-amplified EGFP (GI:1377909) sequence was sub-cloned into pGEM-T Easy vector (Promega) and used to make antisense riboprobe. Hybridization was performed as previously described [27]. The probe was labeled using a DIG labeling kit (Roche) and visualized by NBT/BCIP. Hoechest 33342 was used to stain nuclei. Images were collected using NanoZoomer (Hamamatsu photonics) and a fluorescent microscope (Carl Zeiss AG) with a 5× or 20× objective.

### Statistical analysis

Overall changes in firing rate were evaluated using repeated measures one-way analysis of variance (ANOVA), which was followed up with Bonferroni post-hoc tests. To evaluate whether the laser stimulation affected freezing level, a paired *t-*test was conducted. Data are shown as mean ± standard error. Statistical significance was set at *p* < 0.05.

## Results and Discussion

### Generation of inducible ArchT transgenic mice

To create a transgenic line that allows inducible expression of ArchT in a defined population of neurons, we generated mice carrying TetO-ArchT-GFP transgenes. To examine tetracycline transactivator (tTA)-dependent transgene expression in the mouse brain, we bred the three obtained founder lines with another transgenic mouse line expressing tTA under the control of forebrain-specific α-CaMKII promoter [21]. Consistent with the previously described pattern of tTA expression in the brain [21], all lines of α-CaMKII-tTA × TetO-ArchT-GFP N1 mice displayed similar GFP expression in the forebrain (not shown). We chose the mouse line showing the brightest hippocampal GFP expression for further experimentation.

**Fig 1:**
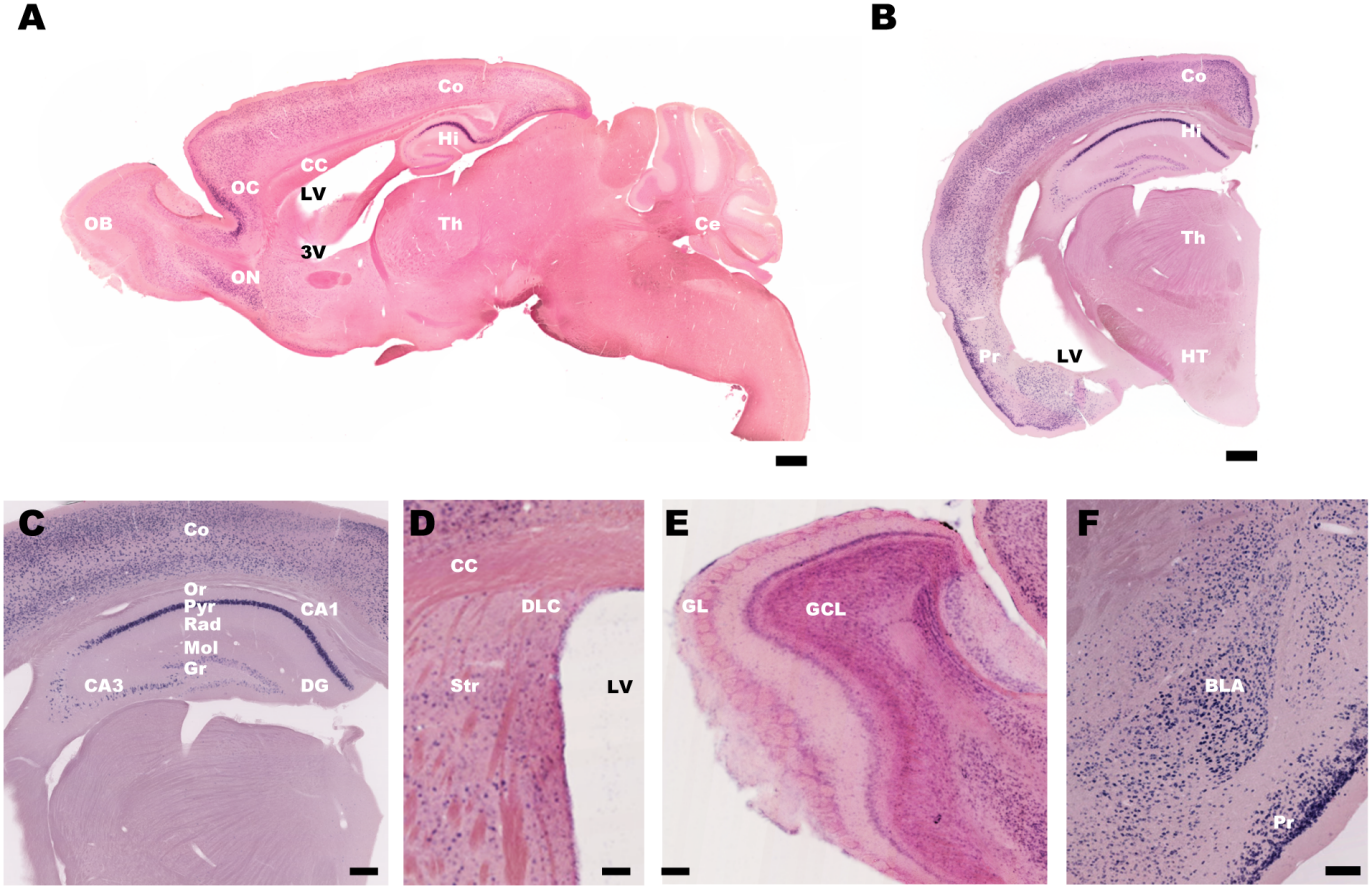
ArchT-GFP mRNA distribution in tTA-CaMKIIα × TetO-ArchT-GFP transgenic mice. *In situ* hybridization of GFP mRNA expression in the brain of tTA-CaMKIIα × TetO-ArchT-GFP transgenic mice. **(A)** Parasagittal and **(B)** coronal sections. Scale bars = 300 μm. Magnified view of the **(C)** hippocampus, **(D)** striatum, **(E)** olfactory bulb, and **(F)** amygdala. Scale bars = 150 μm. The GFP signal is in blue, and the sections were counterstained by Nuclear Fast Red (red). OB: olfactory bulb; OC: orbital cortex; ON: olfactory nucleus; CC: corpus callosum; LV: lateral ventricle; 3V: the third ventricle; Co: cortex; Hi: hippocampus; Th: thalamus; Ce: cerebellum; HT: hypothalamus; DG: dentate gyrus; Or: stratum oriens; Pyr: stratum pyramidale; Rad: stratum radiatum; Mol: molecular cell layer; Gr: granule cell layer; DLC: dorso-lateral corner; Str: striatum; GL: glomerular layer; GCL: granule cell layers; BLA: basolateral amygdala; Pr: piriform cortex.

*In situ* hybridization revealed that transgene mRNA was widely expressed in the forebrain, including the cerebral cortex, hippocampus, striatum, olfactory bulb, and amygdala (Fig 1A–1F), consistent with the reported distribution of tTA in the driver line [21]. In the hippocampus, strong mRNA expression was observed in CA1 pyramidal and subicular neurons, whereas expression in the CA3 and dentate gyrus was notably weaker (Fig 1B and 1C). In the CA1 region, mRNA-expressing cells were restricted to the stratum pyramidale and not found in other layers (Fig 1C), consistent with its expression in excitatory but not inhibitory neurons.

**Fig 2:**
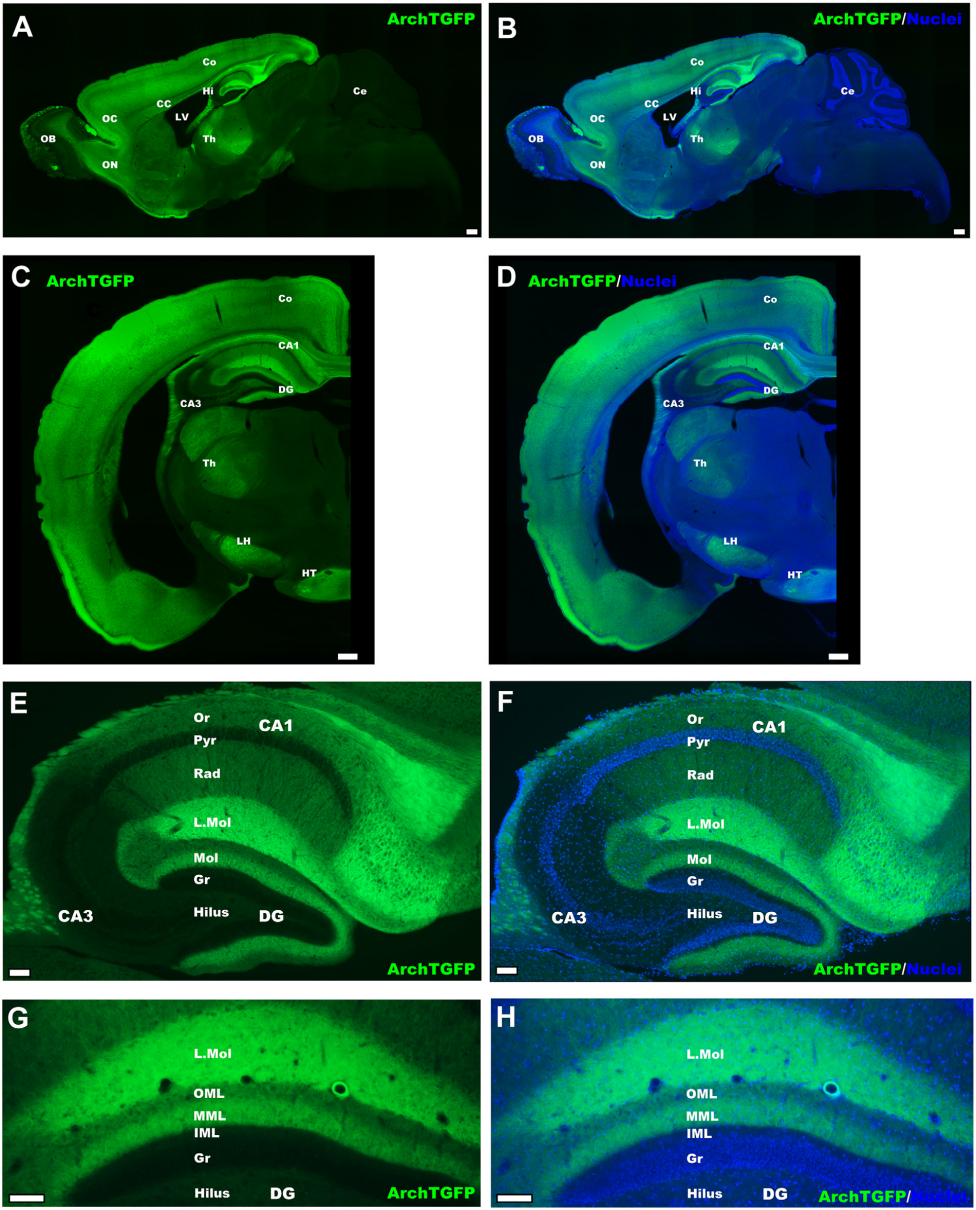
ArchT-GFP expression visualized by GFP native fluorescent signal. ArchT-GFP protein expression in the forebrain of tTA-CaMKIIα × TetO-ArchT-GFP mice. Sagittal view **(A)** without or **(B)** with nuclei staining. Coronal view **(C)** without or **(D)** with nuclei staining. Scale bars = 300 μm. Magnified view of the hippocampus in sagittal view **(E)** without or **(F)** with nuclei staining. Distribution of GFP signal in different layers of the dentate gyrus **(G)** without or **(H)** with nuclei staining (coronal view). Scale bars = 150 μm. LH: lateral hypothalamus; L. Mol: stratum lacunosum-moleculare; OML: outer molecular layer; MML: middle molecular layer; IML: inner molecular layer. See Fig 1 legend for other abbreviations.

Consistent with the expression of mRNA, GFP fluorescence was widely observed in the forebrain (Fig 2A–2D). In the hippocampal CA1 region, a strong signal was observed in the stratum oriens, stratum radiatum, and stratum lacunosum-moleculare. The stratum lacunosum-moleculare showed a particularly strong signal, which continued to the middle molecular layer of the dentate gyrus (Fig 2C–2H). Given the relative scarcity of *in situ* hybridization signal in the dentate gyrus (Fig 1C), this likely represents afferent axonal fibers from the entorhinal cortex, which showed a positive signal. The signal in CA3 region was weaker than that in the CA1 region (Fig 2C–2F). In the absence of tTA, no leaky GFP expression was observed (not shown).

### Inhibition of hippocampal neuronal activity in freely moving mice

**Fig 3:**
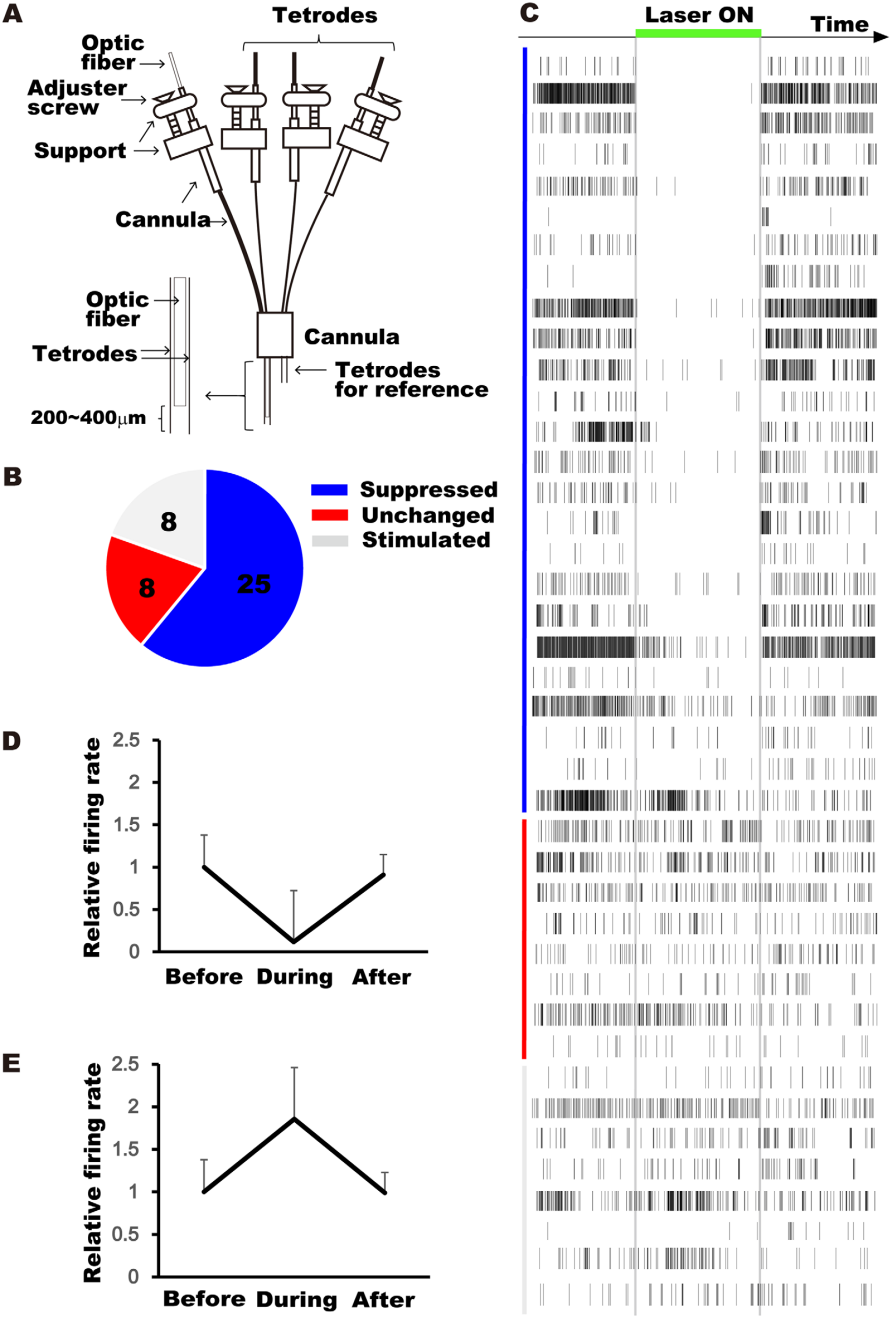
Optogenetic control of neuronal activity in the CA1 of freely moving tTA-CaMKIIα × TetO-ArchT mice. **(A)** Optic fiber and tetrode bundle assembly for simultaneous optical control and electrophysiological recording of neuronal activity. **(B)** Number of isolated units in the CA1 that showed suppression (blue), no change (red) or increased activity (gray) during light delivery. **(C)** Time-dependent activity of all isolated units. Individuals units (rows, *n* = 41) are sorted according to their level of firing rate suppression, from high (top) to low (bottom) (color-coded as in **(B)**). The light illumination period (532-nm, 1 min) is indicated by a green bar and gray vertical lines. **(D)** Average firing rate before, during, and after light illumination for the 25 units that showed suppression. **(E)** Average firing rate before, during, and after light illumination for the 8 units that showed an increase in activity.

To validate ArchT functionality, we performed simultaneous optical illumination and electrical recording in the hippocampal CA1 region of awake α-CaMKII-tTA × TetO-ArchT-GFP mice using a chronically implanted optrode microdrive [13,28,29] (Fig 3A). As the mice were engineered for inducible expression of ArchT in excitatory pyramidal neurons, we isolated a total of 41 units that appeared to be pyramidal neurons based on their spike shape (typically with a width greater than 0.3 ms and an asymmetric peak-trough waveform). Among the isolated units, 25 showed a suppression of action potential frequency during the 1-min period of light delivery, 8 showed an increase in action potential frequency, and the remaining 8 showed no apparent change in firing rate (Fig 3B and 3C). In the cells that showed suppression, the change in firing rate was time-locked and robust (Fig 3D; average suppression: 89% ± 2.6 SE, *F*(2,48) = 11, *p* < 0.0001). The cells that showed an increased firing rate also showed time-locked action potentials (Fig 3E, average increase: 120% ± 39 SE, *F*(2,14) = 3.9, *p* < 0.05). This increase in firing rate may be due to either a homeostatic mechanism induced by changes in local circuit activity [30] or a lack of inhibition from local interneurons that normally receive tonic activation from excitatory neurons. The cells that did not respond to light were considered to be either out of range of the effective light power or to have a low expression of ArchT. Together, these results demonstrate the utility of ArchT for silencing CA1 pyramidal neurons *in vivo*.

### Inhibition of memory recall by ArchT activation in the mouse hippocampus

**Fig 4:**
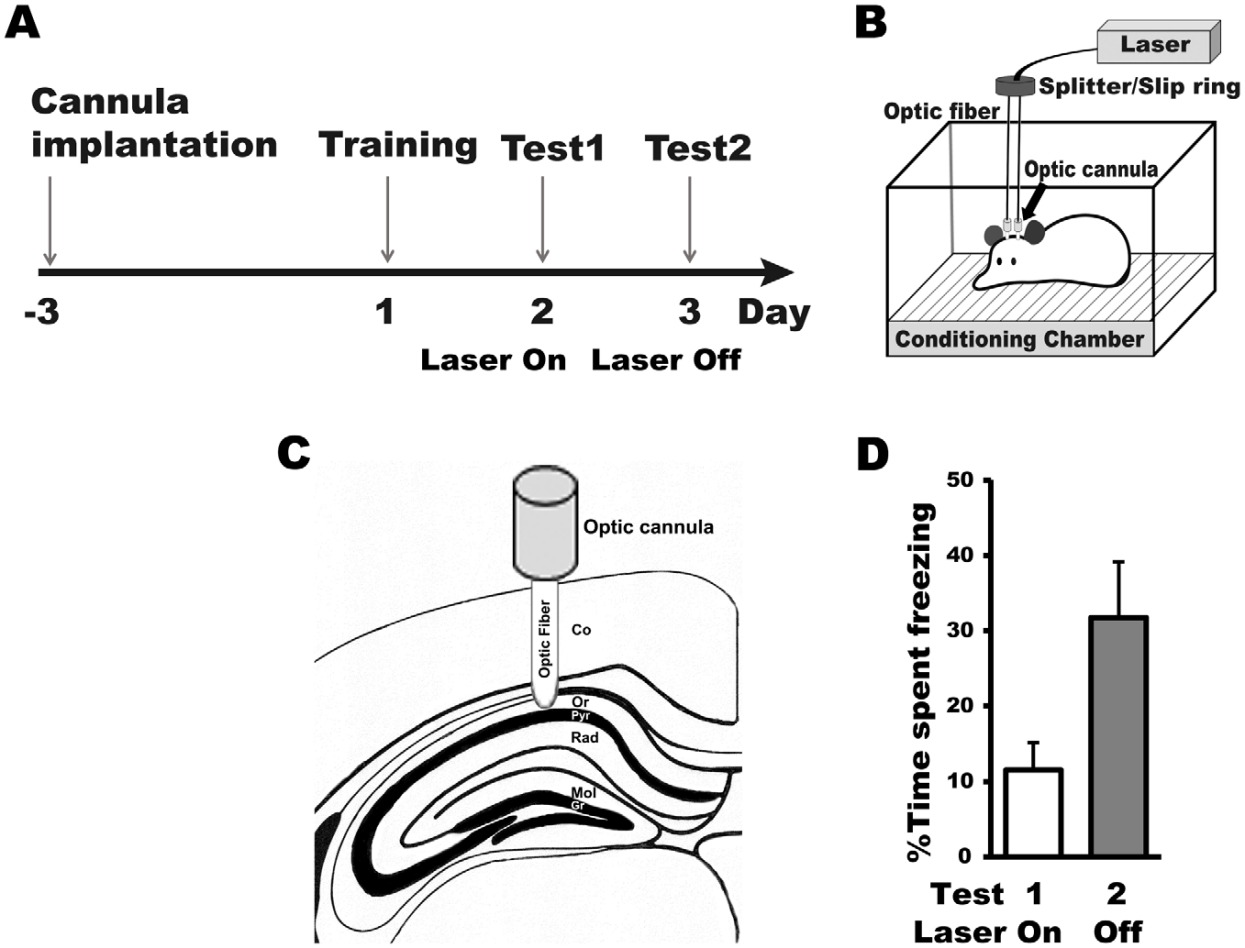
Optogenetic inhibition of CA1 neuronal activity prevented recall of contextual fear memory in tTA-CaMKIIα × TetO-ArchT mice. **(A)** Experimental timeline. Following contextual fear conditioning (i.e., training), light was delivered to the CA1 during test 1 but not during test 2. Experimental setting for **(B)** fear conditioning and **(C)** laser delivery. **(D)** tTA-CaMKIIα × TetO-ArchT mice showed more freezing during test 2 than during test 1, indicating that inhibition of CA1 neuronal activity transiently prevented the recall of a contextual fear memory. Co: cortex; Or: stratum oriens; Pyr: stratum pyramidale; Rad: stratum radiatum; Mol: molecular cell layer; Gr: granule cell layer

Finally, to examine whether α-CaMKII-tTA × TetO-ArchT-GFP mice could be used in memory research, we tested whether shining a 532-nm light on the hippocampus of these mice via an optic cannula inhibited recall of a contextual fear memory. After mice were implanted bilaterally with optic cannulae, they underwent contextual fear conditioning with a single foot shock (Fig 4A and 4B). On two consecutive days after training, the mice were returned to the conditioning chamber for memory tests, during which their freezing behavior was quantified as a measure of memory recall.

During the first test, the optic fibers were connected to the cannulae, and green light was delivered bilaterally to the hippocampi to inhibit neuronal activity (Fig 4C). Mice (*n* = 10) showed little freezing behavior during the test (Fig 4D). During the second test, the optic fibers were again connected to the cannulae, but no light was delivered. This time, mice showed freezing behavior (paired *t*-test, *t*(9) = 3.5, *p* < 0.005), indicating the presence of a contextual fear memory (i.e., association between the context and foot shock). These results suggest that the absence of freezing during the first test was not due to a permanent loss of memory. Rather, inhibition of hippocampal neuron activity produced a transient deficit in memory recall, possibly due to a disruption of memory storage or retrieval as described in previous studies [1,10].

In conclusion, our results demonstrate that TetO-ArchT-GFP transgenic mice enable the reversible inhibition of neuronal activity with temporal precision in a genetically defined population of neurons. The use of these mice can advance memory research by allowing a strong causal link to be made between the activity of target neurons and information processing *in vivo*.
